# Structural Properties and Antifungal Activity against *Candida albicans* Biofilm of Different Composite Layers Based on Ag/Zn Doped Hydroxyapatite-Polydimethylsiloxanes

**DOI:** 10.3390/polym8040131

**Published:** 2016-04-09

**Authors:** Andreea Groza, Carmen Steluta Ciobanu, Cristina Liana Popa, Simona Liliana Iconaru, Patrick Chapon, Catalin Luculescu, Mihai Ganciu, Daniela Predoi

**Affiliations:** 1 National Institute for Laser, Plasma and Radiation Physics, 409 Atomistilor St. P.O. BOX MG 36, Magurele 077125, Romania; andreea@infim.ro (A.G.); catalin.luculescu@inflpr.ro (C.L.); ganciu@infim.ro (M.G.); 2 National Institute for Materials Physics, 405 A Atomistilor Street, P.O. Box MG 07, Magurele 077125, Romania; ciobanucs@gmail.com (C.S.C.); cristina.lyana@gmail.com (C.L.P.); simonaiconaru@gmail.com (S.L.I.); 3 Faculty of Physics, University of Bucharest, 405 Atomistilor Street, P.O. Box MG1, Magurele 077125, Romania; 4 Horiba Jobin Yvon S.A, 16-18, rue du Canal, Longjumeau Cedex 91165, France; patrick.chapon@horiba.com

**Keywords:** composite layers, Ag/Zn doped hydroxyapatite, PDMS, *C. albicans* biofilm

## Abstract

Modern medicine is still struggling to find new and more effective methods for fighting off viruses, bacteria and fungi. Among the most dangerous and at times life-threatening fungi is *Candida albicans*. Our work is focused on surface and structural characterization of hydroxyapatite, silver doped hydroxyapatite and zinc doped hydroxyapatite deposited on a titanium substrate previously coated with polydimethylsiloxane (HAp-PDMS, Ag:HAp-PDMS, Zn:HAp-PDMS) by different techniques: Scanning Electron Microscopy (SEM), Glow Discharge Optical Emission Spectroscopy (GDOES) and Fourier Transform Infrared Spectroscopy (FTIR). The morphological studies revealed that the use of the PDMS polymer as an interlayer improves the quality of the coatings. The structural characterizations of the thin films revealed the basic constituents of both apatitic and PDMS structure. In addition, the GD depth profiles indicated the formation of a composite material as well as the successful embedding of the HAp, Zn:HAp and Ag:HAp into the polymer. On the other hand, *in vitro* evaluation of the antifungal properties of Ag:HAp-PDMS and Zn:HAp-PDMS demonstrated the fungicidal effects of Ag:HAp-PDMS and the potential antifungal effect of Zn:HAp-PDMS composite layers against *C. albicans* biofilm. The results acquired in this research complete previous research on the potential use of new complex materials produced by nanotechnology in biomedicine.

## 1. Introduction

In an era when science and technology have reached a point when the impossible was transformed into reality, when cars learn to drive themselves and intelligent robots learn how to walk as human beings and to interpret human emotions, the medical field is still struggling to find new and more effective methods for fighting off viruses, bacteria and fungi. Out of almost 611,000 species of fungi, only 600 species are considered to be human pathogens [1,2,3]. Among the most dangerous and at times life-threatening fungi is *Candida albicans*, which in the United States is considered to be the fourth most frequently encountered cause of systemic infections acquired from hospitals [1,4,5]. This was possible due to the increasing resistance to antibiotics of different types of fungi and also due to the limited number of antifungal drugs. *Candida* species is one of the most dangerous human fungal pathogens responsible for deep tissue and mucosal infections, especially in the oral cavity. In addition, it was proved that up to 50% of hospital-related *C. albicans* infections are deadly [1,4,5]. According to Samaranayake *et al.* [6] infections caused by *Candida* species are associated to biofilm growth. In this context, it is imperative to find new solutions for fighting off the infections caused by this fungus.

Although nanotechnology has brought improvements to the field of nanomedicine, providing improved materials that are able to mimic the human body tissues to a certain point, there are still many challenges to overcome. One of the medical fields that has been improved in the last decades due to the advances made in the area of biomaterials is the field of orthopedics. In order to improve the metallic prosthesis that are used in orthopedic surgeries, different biocompatible materials have been used as coatings. In this context, a great deal of attention was directed to bioceramics. Their ability to promote new bone formation and their capacity to facilitate the adherence to tissues as well as to induce growth of peripheral tissues [7] have made them excellent candidates for various applications in the biomedical field. In this context, synthetic hydroxyapatite (HAp) has attracted the attention of researchers and medics alike due to its similarity to the inorganic component of the human bone [8]. Currently it is used as adsorbent for different proteins and enzymes [8] or as hard tissue substitute in orthopedic and dental surgeries [9]. Hydroxyapatite is used in different shapes and sizes, from granules, porous or solid matter to fine nanoparticles [8]. However, although hydroxyapatite has remarkable biocompatible and bioactive properties [10,11,12], it was demonstrated that HAp coatings promote bacterial growth along with its evident osteoconductivity [10,13]. It was observed that coated devices are more prone to peri-implantitis than uncoated ones [10,13].

A solution to increase the antimicrobial activity of hydroxyapatite based coatings was to dope HAp with different metal ions which possess special antimicrobial properties. In this context, a great deal of attention was directed to silver and zinc ions. Silver (Ag) is a well-known biocidal agent used in various medical fields, which succeeds in eradicating a large number of bacteria and fungi [14,15]. According to previous research [14,16], silver disrupts the respiratory function of bacteria by attaching to the bacterial cell membrane and modifying its permeability [14,16]. Another explanation for its ability to destroy bacteria is that very small Ag nanoparticles have the capacity to penetrate the bacterial cell and to bind with their DNA [14,15,17,18]. Zinc (Zn) is one of the trace elements which is naturally found to some extant in the composition of the human hard tissue (in the enamel of the teeth and in the bone tissue) [19,20]. According to several researchers, zinc could be considered one of the most important element in medicine due to its vital role in the proper functioning of almost 200 enzymes [19,21]. Doping HAp with Zn^2+^ ions was found natural also due to its ability to promote bone formation [22,23]. Therefore, embedding either silver or zinc ions in the structure of synthetic hydroxyapatite leads to a superior material more adequate for being used in different medical applications. Another drawback of hydroxyapatite is its brittleness [24,25]. In order to improve the brittle nature of HAp, scientists have found a solution by combining it with various biopolymers. Polydimethylsiloxane (PDMS) has attracted the attention of scientists due to its biocompatibility. It has been used in various biomedical implants since the 1960s [26]. Different works demonstrated that most of PMDS remarkable properties arise from the siloxane bond [27,28,29,30]. Among these properties, its low glass transition temperature at −123 °C, hydrophobicity, and its low surface energy, its chemical and thermal stability as well as low electrical conductivity, physiological inertness and its high permeability to gasses [27,28] are some of its advantages. Recently, Yaling Lin *et al.* [31] revealed the antimicrobial activities of polysiloxane-containing quaternary ammonium salts against bacteria and phytopathogenic fungi, considering that polysiloxanes proved high static and dynamic flexibility in many solvents, high permeability and special surface properties. In addition, PDMS layers became a preferred soft substrate for culturing different types of cells [32] due to their biocompatibility [33], non-toxicity toward many species of organisms and their biodegradability [34].

Different techniques like: sol–gel methods, radio-frequency magnetron sputtering plasma-spray technique, pulsed laser deposition or electrodeposition are now used for the deposition of amorphous HAp coatings on different types of substrates [35,36]. As these coatings are further crystallized at elevated temperatures, during these thermal treatments, many cracks are formed, as the bonding strength of the coating layer with the substrate is affected due to the thermal expansion mismatch between the coating and the metal substrate [36]. As a result, different types of interlayers are now used as reinforcement agents for improving the delamination of HAp coatings. As an example, due to their own excellent compatibility with the living tissues, and their high chemical inertness [37] SiO_2_ layers have been used for this purpose.

In this context, in our previous papers [38] we analyzed the physico-chemical influences of a polydimethylsiloxane film used as interlayer for an Ag:HAp coating. Given that the X-ray diffraction spectra evidenced the crystalline form of the hydroxyapatite doped with silver in the Ag:HAp-PDMS composite layer and the Fourier Transform Infrared Spectroscopy measurements indicated the SiO_4_^4−^ ions formation, we supposed that the SiO_4_^4−^/PO_4_^3−^ ions substitution mechanism is possible to take place. In addition, it was shown [39] that the hardness (H) of the Ag:HAp layer increased in the presence of the PDMS layer while its Young’s modulus (Y) decreased.

The goal of this research was an *in vitro* evaluation of the effects of various composite layers based on silver or zinc doped hydroxyapatite/polydimethylsiloxane (Ag:HAp-PDMS and Zn:HAp-PDMS) or hydroxyapatite/polydimethylsiloxane (HAp-PDMS) on *Candida albicans* (*C. albicans*) adhesion to layers surfaces. These composite layers that cover commercially titanium substrates were prepared by combining corona discharge at atmospheric pressure and sol-gel dip coating method.

As a result of the fact that titanium-based implants could potentially be responsible *C. albicans* for infections [40,41], our studies presented in this work may bring new specific information on the interactions between fungal cells and complex layers (Ag:HAp-PDMS, Zn:HAp-PDMS and HAp-PDMS). Despite the fact that the antimicrobial properties of silver and zinc have been extensively studied, scientific knowledge of the complex interactions between complex layers based on silver and zinc are very limited.

In this context, the research has focused on studies of the Ag:HAp-PDMS, Zn:HAp-PDMS and HAp-PDMS complex layers surface and structure. These composite layers deposited on Ti substrates were analyzed by different techniques such as Scanning Electron Microscopy (SEM), Glow Discharge Optical Emission Spectroscopy (GDOES) and Fourier Transform Infrared Spectroscopy (FTIR). Furthermore, the *in vitro* evaluation of the antimicrobial effect of the various complex layers, which appeared to be active against *C. albicans* biofilm embedded cells at distinct intervals of time, is presented.

## 2. Materials and Methods

### 2.1. Materials

Calcium nitrate tetrahydrate [Ca(NO_3_)_2_∙4H_2_O, Aldrich St. Louis, MO, USA], phosphorus pentoxide (P_2_O_5_, 98%, Merck, Kenilworth, NJ, USA), silver nitrate (AgNO_3_, 99.9%, Alfa Aesar, Ward Hill, MA, USA) and zinc nitrate hexahydrate (Zn(NO_3_)_2_∙6H_2_O, 99%, Alfa Aesar, Ward Hill, MA, USA) were used as precursors for the synthesis of HAp, Ag:HAp and Zn:HAp thin films. All the precursors were used without further purification. Vinyl polydimethylsiloxane (PDMS, *M*_w_ ≈ 25,000, Sigma Aldrich, St. Louis, MO, USA) liquids were used as precursors for PDMS layers generation. Commercially pure titanium (Ti, 1.0 mm thick, Alfa Aesar, Ward Hill, MA, USA) foil was cut into disks with a 20 mm diameter and were used as substrates. All the disks were degreased (five time) in an ultrasonic bath with acetone. After each degreasing the disks were rinsed with deionized water.

### 2.2. Deposition of PDMS LAyers on Ti Substrates

The PDMS layers were deposited on Ti substrates in atmospheric air pressure corona discharges in a point to plane electrode configuration. Under corona charge injection (mainly negative oxygen ions) the liquid precursor of vinyl terminated polydimethylsiloxanes that lies on the anode (Ti substrate), is transformed into a solid polymer layer after two hours. The entire deposition procedure was described in detail in [42,43].

### 2.3. Deposition of HAp Layers on a Titanium Substrate Previously Coated with a PDMS Layer

Hydroxyapatite was synthesized by sol-gel method using calcium nitrate tetrahydrate (Ca(NO_3_)_2_∙4H_2_O) and phosphorus pentoxide (P_2_O_5_) as Ca and P precursors. On the one hand, a designed amount of Ca(NO_3_)_2_∙4H_2_O was dissolved in anhydrous ethanol. On the other hand, a proper amount of P_2_O_5_ was dissolved similarly in anhydrous ethanol. The phosphorus solution was added drop by drop into the Ca solution under vigorous stirring. The obtained solution was stirred for 30 min at 40 °C. The resulted sols were aged for 72 h. Finally, the obtained sols were clear and stable. Furthermore, the titanium substrate previously coated with a PDMS layer was immersed into HAp sol. Each coating was dried at 80 °C for 30 min and the coating procedure was repeated five times. In the end the samples were thermally treated at 600 °C for 2 h in air, with a heating rate of 10 °C/min [44].

### 2.4. Deposition of Ag:HAp and Zn:HAp Layers on a Titanium Substrate Previously Coated with a PDMS Layer

The composition ratios in the Ag:HAp (Ca_10−x_Ag_x_(PO_4_)_6_(OH)_2_, *x*_Ag_ = 0.3) and Zn:HAp (Ca_10−x_Zn_x_(PO_4_)_6_(OH)_2_, *x*_Zn_ = 0.3) sols were adjusted so that the [Ca + Ag]/P and [Ca + Zn]/P ratios would be equal to 1.67 [45,46,47].

In order to obtain Ag:HAp, silver nitrate was used for the substitution of Ca with Ag ions in the hydroxyapatite structure. Firstly, AgNO_3_ and Ca(NO_3_)_2_∙4H_2_O were mixed and dissolved in anhydrous ethanol. On the other hand, a proper amount of P_2_O_5_ was dissolved similarly. The phosphorus solution was added drop by drop to the (Ag + Ca) solution under vigorous stirring. The obtained solution was stirred for 30 min at 40 °C. The obtained sols were aged for 72 h. Finally, the obtained sols were clear and stable. Furthermore, the titanium substrate previously coated with a PDMS layer was immersed into the Ag:HAp sol. Each coating was dried at 80 °C for 30 min and the coating procedure was repeated five times. In the end the samples were thermally treated at 400 °C for 2 h in air with a heating rate of 10 °C/min [39,48].

The same procedure was followed for obtaining Zn:HAp sol. This time, for the substitution of Ca ions with Zn ions in the structure of HAp, zinc nitrate hexahydrate (Zn(NO_3_)_2_∙6H_2_O), was used.

### 2.5. Structural Characterizations

Roughness measurements of the Ti substrates were performed using a Mahr perthometer (Göttingen, Deutschland). For each Ti substrate the arithmetic mean deviation *R*_a_ of roughness profile, mean peak to valley height *R*_z_ and root mean square deviation *R*_q_ of the roughness profile were determined.

The morphological features of the hydroxyapatite coating surfaces were investigated by Scanning Electron Microscopy (SEM) using a FEI Inspect S scanning electron microscope (Hillsboro, OR, USA) in both high- and low-vacuum modes.

The elemental depth profile analysis of the Ag:HAp-PDMS, Zn:HAp-PDMS and HAp-PDMS coatings were performed by Glow Discharge Optical Emission Spectroscopy (Horiba Company, Longjumeau, France). The experimental conditions used for the operation of the GD Profiler were: 650 Pa, 35 W RF power impulse mode at1kHz and a duty cycle of 0.25.

The IR spectra of the Ag:HAp-PDMS, Zn:HAp-PDMS and HAp-PDMS coatings obtained on Ti substrate were acquired using a SP100 IR Perkin Elmer spectrometer (Waltham, MA, USA) equipped with a variable angle specular reflectance accessory. The measurements were carried out for an angle of reflection of 300. According to [10], the second derivative IR spectra were acquired after performing a 5-point smoothing of the IR absorbance spectra. For the 450–2000 cm^−1^ spectral ranges, the peak fitting analyses were carried out using procedures described by Iconaru et al. [10].

### 2.6. In Vitro Antifungal Activity

The bacterial strain used in the biofilm formation was *C. albicans* ATCC 10231. In order to assess the biofilm formation on the surface on different surface (Ti, PDMS, HAp-PDMS, Zn:HAp-PDMS and Ag:HAp-PDMS) of composite layers, 0.5 McFarland microbial suspension in sterile saline obtained from 24 h microbial cultures were grown on the surface of the thin films in a liquid yeast peptone glucose (YPG) medium. Every 24, 48 and 72 h the thin films were removed from the culture medium. They were washed using sterile saline solution in order to remove the non-adherent microbial cells and reintroduced into sterile saline. After that, the samples were vortexed for suspending the microbial cells embedded in the biofilm formed on the thin films specimens. This entire procedure was repeated for the thin films colonized with fungal biofilms at 48 and 72 h.

The biofilm formation of *C. albicans* on different surface (Ti, PDMS, HAp-PDMS, Zn:HAp-PDMS and Ag:HAp-PDMS) of composite layers, was investigated using confocal laser scanning microcopy (CLSM). For the CLSM observation, the cells were stained for 2 min with propidium iodide, washed 2 times with water, air dried and then visualized in reflection and fluorescence modes by using a TCS SP confocal microscope, equipped with a 10X HCX PL FLUORITE objective, with a numerical aperture NA of 0.3. In order to acquire both reflection and fluorescence, an Ar ion laser (488 nm) was used.

The biofilm morphology of the samples was analyzed using scanning electron microscopy. For SEM observation, the *C. albicans* biofilms were previously fixed on the thin films using 200 μL cold absolute methanol for 5 min. The methanol excess was removed and the films were allowed to air dry. The samples were place on a sample holder using carbon tape and then analyzed.

## 3. Results and Discussions

The results presented in this paper were focused on evaluation of the antifungal activity of different surfaces (Ti, PDMS, HAp-PDMS, Zn:HAp-PDMS and Ag:HAp-PDMS) against *C. albicans* on biofilm development. In addition, we studied the morphological and structural characteristics of different surfaces such as PDMS, HAp-PDMS, Zn:HAp-PDMS and Ag:HAp-PDMS.

### 3.1. Scanning Electron Microscopy

**Figure 1:**
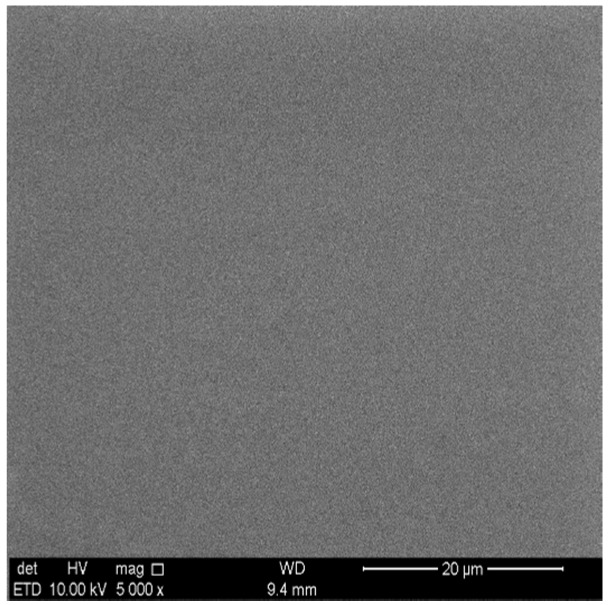
SEM image of Polydimethylsiloxane (PDMS) layer deposited on Ti substrate in negative corona discharge.

Depending on the Ti substrate surface roughness (*R*), the characteristic features of Ag:HAp, Zn:HAp and HAp coatings were analyzed with or without a polymer interlayer by scanning electron microscopy. The morphology of a PDMS layer deposited on a Ti substrate with an optically polished surface, (*R*_a_ = 0.084 μm, *R*_z_ = 0.489 μm, *R*_q_ = 0.108 μm) in the corona discharge in air at atmospheric pressure is shown in Figure 1.

As the polymer covered only a circular area of 10 mm in diameter on the center of the substrate surface and the HAp was deposited on the entire Ti disk surface (20 mm in diameter), the interface zone between the Ag:HAp-PDMS, Zn:HAp-PDMS, HAp-PDMS and Ag:HAp, Zn:HAp, HAp respectively will be further investigated.

**Figure 2:**
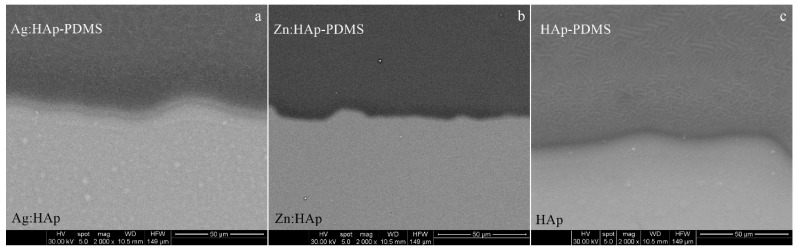
The SEM images of the interface zone between: (**a**) Ag:HAp-PDMS (darker zone) and Ag:HAp (lighter zone); (**b**) Zn:HAp-PDMS (darker zone) and Zn:HAp (lighter zone); (**c**) HAp-PDMS (darker zone) and HAp (lighter zone).

In Figure 2 are shown the images of Ag:HAp-PDMS, Zn:HAp-PDMS and HAp-PDMS layers, deposited on Ti substrates with optically polished surfaces, at their interface with the Ag:HAp, Zn:HAp or HAp layers. The coatings seem to be compact and homogeneous with no cracks, the polymer acting like a matrix that allows the hydroxyapatite embedding.

It is well known that for biological applications, the physical properties of the coatings like: uniformity, delamination or cracking are very important issues that depend not only on deposition procedure but also on the roughness of the substrate surface. A rough substrate surface will determine a non-uniform deposited layer, the possibility of coating delamination and cracking being increased. Therefore, we supposed that the use of a polymer interlayer for the HAp coating could overcome its delamination even when cracks appear. Thus, in the following, we extend our SEM investigations to the case of HAp based coatings deposited on rough surfaces in the presence and the absence of a PDMS layer laying on the substrate surface.

**Figure 3:**
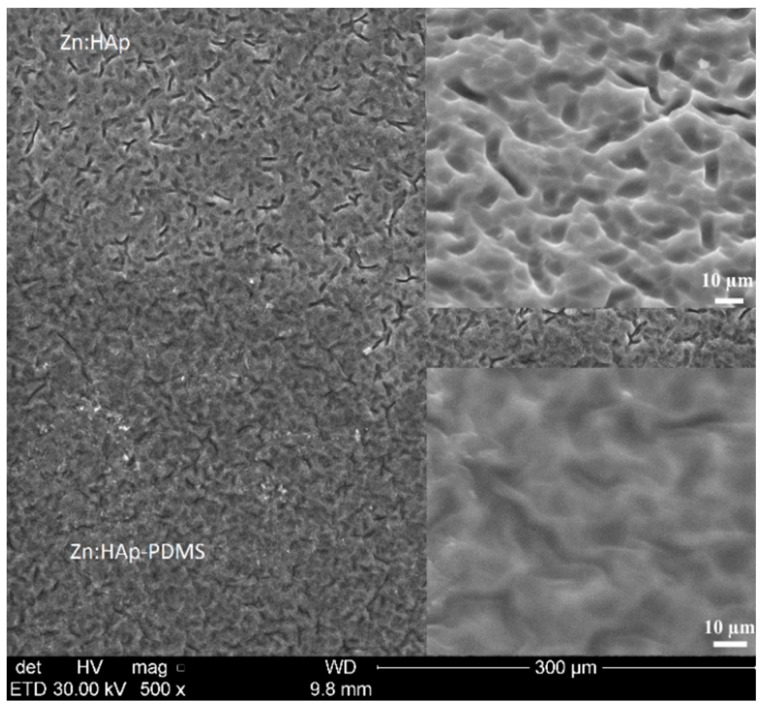
SEM images of the interface zone between Zn:HAp-PDMS and Zn:HAp coating.

On a Ti rough surface (*R*_a_ = 0.520 μm, *R*_z_ = 3.064 μm, *R*_q_ = 0.643 μm) partially coated with a PDMS layer, we deposited by sol-gel dip coating method a Zn:HAp layer. The SEM images presented in Figure 3 indicate the embedding of the Zn:HAp coating into the PDMS layer.

When the sample was tilted at an angle of 45°, (enlarged images from Figure 3), the smoother surface of the Zn:HAp-PDMS was better evidenced in comparison with the Zn:HAp coating. Similar morphologies to the Zn:HAp coatings were obtained for the other two studied samples (Ag:HAp and HAp layers).

**Figure 4:**
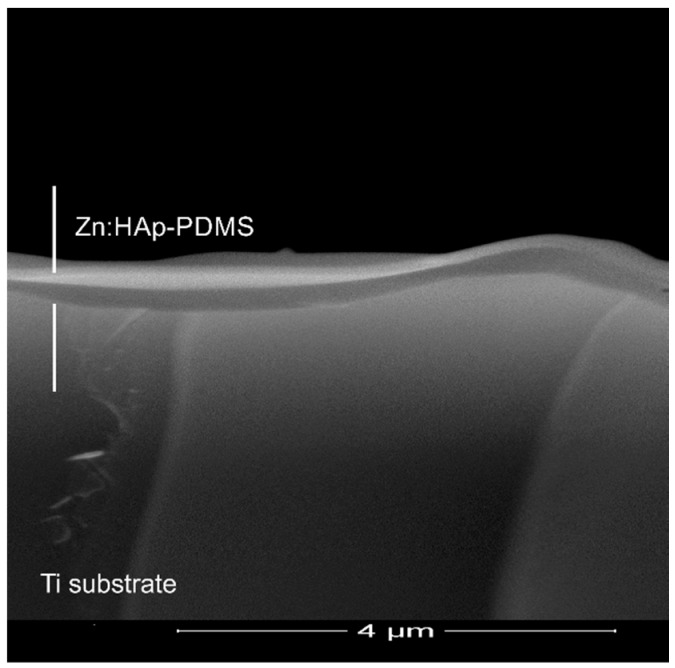
SEM images of the transversal cross section of a Zn:HAp-PDMS layer deposited on a rough Ti surface substrate.

The SEM analysis of the transversal cross section of a Zn:HAp-PDMS layer, Figure 4, indicated its thickness (around 350 nm) as well as the role of the polymer presence on the substrate surface. It could be observed how the polymer covers uniformly the uneven substrate surface. Thus, it could be a promoter for the increase of Zn:HAp coating adherence to the rough substrate.

**Figure 5:**
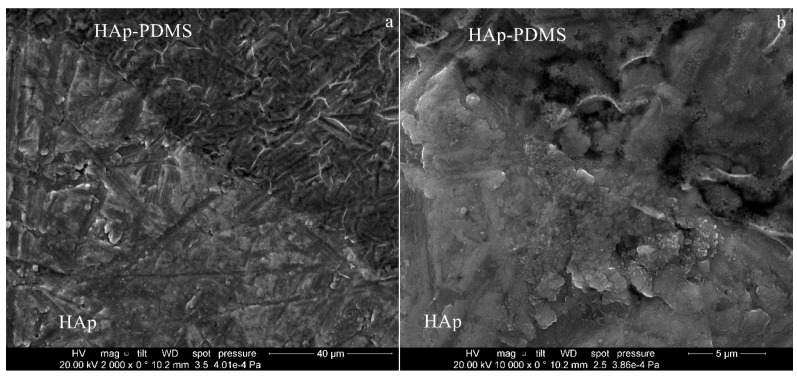
SEM images of the interface zone between HAp-PDMS (darker zone) and HAp (lighter zone) for different magnification: (**a**) ×2000; (**b**) ×10,000.

On a scratched and rough (*R*_a_ = 0.462 μm, *R*_z_ = 2.349 μm, *R*_q_ = 0.550 μm) surface of Ti substrate partially coated with the polymer, we deposited a HAp layer. In Figure 5a,b can be observed that the scratches from the Ti surface affect the HAp coating morphology and layer uniformity, its delamination being promoted. Even if the coating is non-uniform, delaminated and cracked, its embedding into the polymer could prevent it from further deterioration. The Ag:HAp and HAp coatings had similar morphologies when deposited on Ti surfaces.

**Figure 6:**
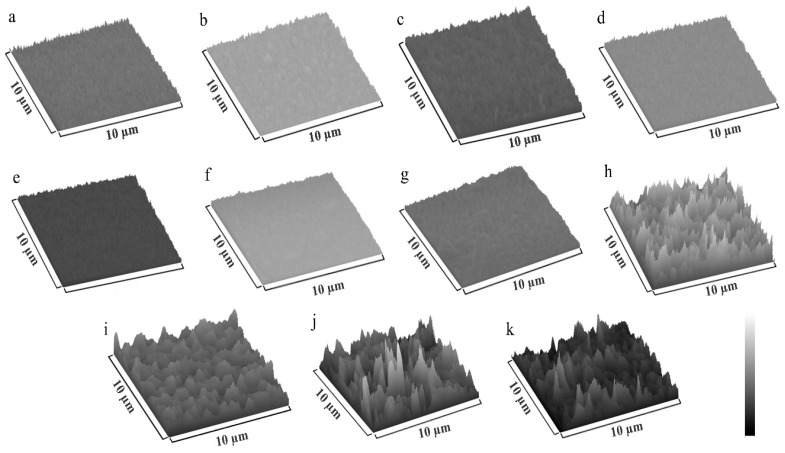
3D surface plot of: (**a**) PDMS (SEM image from Figure 1); (**b**) Ag:HAp (SEM image from Figure 2a); (**c**) Ag:HAp-PDMS layer (SEM image from Figure 2a); (**d**) Zn:HAP (SEM image from Figure 2b); (**e**) Zn:HAP-PDMS (SEM image from Figure 2b); (**f**) HAp (SEM image from Figure 2c); (**g**) HAp-PDMS (SEM image from Figure 2c); (**h**) Zn:HAp (SEM image from Figure 3); (**i**) Zn:HAp-PDMS (SEM image from Figure 3); (**j**) HAp (SEM image from Figure 5a); (**k**) HAp-PDMS (SEM image from Figure 5a).

The texture of Ag:HAp, Zn:HAp and HAp coatings deposited on mirror like surfaces, in the presence and absence of the polymer layer, was obtained performing a 3D surface plot (Figure 6) of their SEM images using a processing image using Image J software (https://imagej.nih.gov/ij/).

### 3.2. Glow Discharge Optical Emission Spectrometry (GDOES)

**Figure 7:**
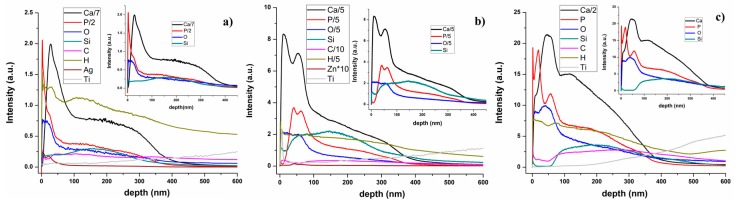
GDOES depth profiles of: (**a**) Ag:HAp-PDMS; (**b**) Zn:HAp-PDMS; (**c**) HAp-PDMS composite layers.

Glow discharge optical emission spectrometry (GDOES) is a chemical analytical method often used for the evaluation of constituent elements distributed throughout the coatings. It gives information on a macroscopic scale (usually the diameter of the investigated surface is around 4 mm) about the depth profiling of thin films that cover metallic surfaces, having short measuring time and high sensitivity. In GDOES, the emission intensities of the elements contained in a coating are measured as function of the sputtering time of the sample in a glow discharge plasma. GDOES depth profiles of the chemical elements contained in the Ag:HAp, Zn:HAp, and HAp films deposited on substrates previously covered with a PDMS layer are presented in Figure 7.

The Ca and P depth profiles, the main elements in a HAp based coating, had similar behavior in all investigated samples. According to our previous studies [42], in a GDOES depth profile spectrum of a PDMS layer, we marked the surface and the polymer/substrate interface by the Si depth profile curve behavior. The Si, Ca and P depth profile curves begin and ended simultaneously, thus indicating that the HAp was embedded into the polymer.

The broadening of the depth profile curves associated with the chemical elements identified in the spectra from Figure 7 were almost the same in all the samples, being no clear delimitation between them as in the case of multilayer coatings. Different reasons like roughness of the layer/substrate interface as the signals recorded by the GD Profiler are averaged over the investigated zone, crater bottom flatness or a composite material, could determine such of behavior. Since the substrates are mirror like surfaces and the operating conditions were chosen for providing a flat crater bottom, the GD depth profiles from Figure 7 seem to indicate the formation of a composite material.

By reaching the coating/substrate interface, the steep rise in the Ti depth profile curve is accompanied by the decrease of all signals specific to the elements contained in the investigated sample [42].

### 3.3. Fourier Transform Infrared Spectroscopy (FT-IR)

**Figure 8:**
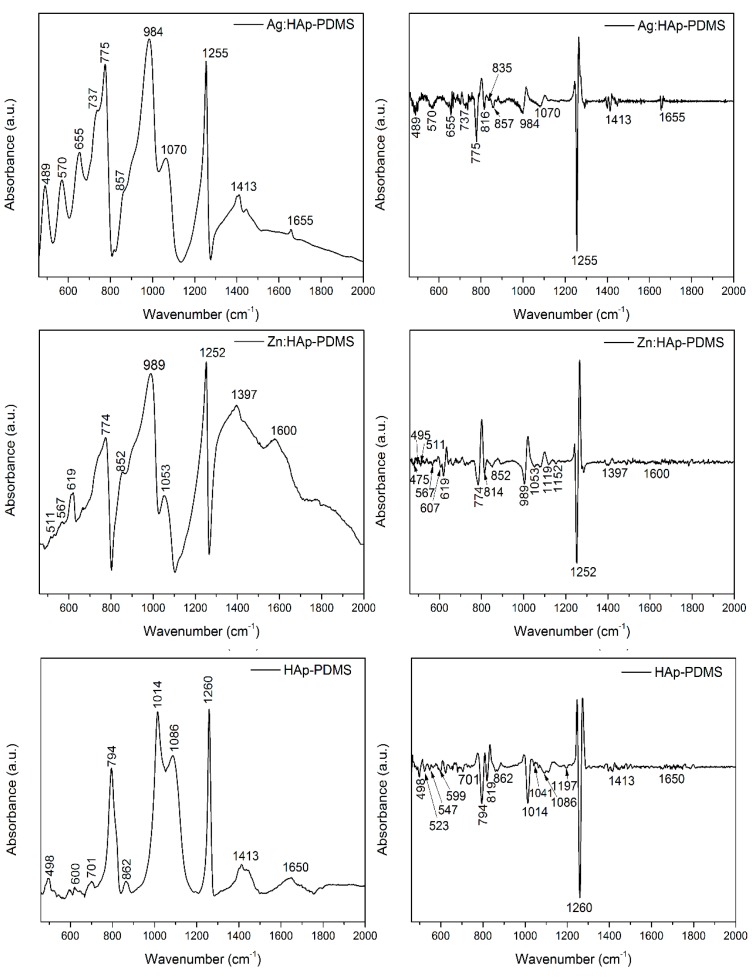
FTIR spectra and second derivatives of the HAp-PDMS, Ag:HAp-PDMS and Zn:HAp-PDMS composite layers.

The IR spectra of the HAp-PDMS, Ag:HAp-PDMS and Zn:HAp-PDMS layers that cover uniformly the mirror like surfaces of Ti substrates were obtained by reflectance spectroscopy. The FTIR absorbance spectra together with their second derivative (in the spectral region of 450–2000 cm^−1^) are presented in Figure 8. In the IR absorbance spectra of the studied samples, the main vibrational bands were attributed to the Si–O–Si (about 1004 and 1071 cm^−1^) [49], SiO_4_^4−^ (around 490 and 695 cm^−1^) [44,48,50,51,52,53,54], Si–O and Si–C (approximatively 862 cm^−1^) [55] functional groups present in the PDMS layer. In our previous studies [42,43], we showed that during the polymerization time in negative corona discharge, in a PDMS layer, SiO_2_ like structures were generated, the SiO_4_^4−^ groups being predominant at the polymer surface. As a result of the thermal treatment performed on the samples after the deposition of HAp, Ag:HAp and Zn:HAp on the titanium substrates previously coated with the PDMS layer, some substitutions of the PO_4_^3−^ from the apatitic structure by SiO_4_^4−^ were possible. [38,52,53,56,57,58]. Furthermore, the appearance of SiO_4_^4−^ groups in the spectral region of 450–650 cm^−1^ at 498 (in HAp-PDMS layer) respectively 511 cm^−1^ (in Zn:HAp-PDMS layer), was previously attributed to a partial loss of phosphate groups and/or of the symmetry at the site caused by substitution of silicate species in Si-HAp [51,59,60].

The IR bands identified at about 600 and 1053 cm^−1^ correspond to the PO_4_^3−^ groups that ascertain the HAp structure [48]. The characteristic peaks associated to the H_2_O, OH, Si–OH vibrations were identified near 1600 cm^−1^ [61], while the peak from 1260 cm^−1^ is often attributed to the C–H bond from the Si–CH_3_ group [62]. On the other hand, the vibrational bands found at 700 respectively 737 cm^−1^ are specific to C–H vibration in CH_3_ groups that belong to Si–CH_3_ [49,63].

In Table 1 are summarized the main IR vibrational bands observed in the FTIR spectra of HAp-PDMS, Ag:HAp-PDMS and Zn:HAp-PDMS composite layers.

A powerful tool usually used for the determination of the weak absorption bands and for improvement of the resolution of overlapped bands is the second derivative of FTIR spectra. The second derivative spectrum of each sample is presented in the Figure 8.

The formation of SiO_4_^4−^ groups [44,48,50,51,52,53,54] was also evidenced in the second derivative spectrum of the investigated samples. Furthermore, in the case of HAp-PDMS layer, the presence of bands characteristics to the ν_4_ PO_4_^3−^ vibrations was noticed. These bands are characteristic to the hydroxyapatite structure and are located at 547 and 599 cm^−1^ respectively [10,48]. The IR band which appears in the second derivative spectra of HAp-PDMS at 1041 cm^−1^ is attributed to ν_3_ PO_4_^3−^ from the HAp structure [10,48]. Moreover, in the same spectra several bands at 523 [51,59,60] (characteristic to the SiO_4_^4−^ groups), 819 (arisen from the Si–O–Si bonds) [51,52,64] and at 1197 cm^−1^ (attributed to the S–O bonds) were observed [42,51,59]. Additionally, in the second derivative spectra of Ag:HAp-PDMS composite layer the band from 835 cm^−1^ was identified as belonging to Si–O–Ag bonds [65,66].

The second derivative spectrum of Zn:HAp-PDMS composite layer revealed the presence of vibrational bands characteristic to Si–O–Si bonds (at 814 cm^−1^) [55,64], Si–O bonds (1119 and 1152 cm^−1^) [42,54,63] as well as the ν_4_ (at 607 cm^−1^) [10,38], ν_2_ (at 475 cm^−1^) [10,44] and ν_3_ vibrations (at 1053 cm^−1^) [10,48] of the PO_4_^3−^ functional group. The band registered at 495 cm^−1^ [38] is attributed to the SiO_4_^4−^ functional groups. From the second derivative spectra result that the Si–O and P–O, IR bands present in the Ag:HAp-PDMS, Zn:HAp-PDMS and HAp-PDMS composite layers are overlapping.

Consequently, the appearance respectively the absence of the certain IR bands characteristic to P–O bonds from the absorbance spectra of the investigated films as result of Si–O/P–O bands overlapping, could indicate the embedding of the Ag:HAp, Zn:HAp and HAp into the PDMS layer. Some previous studies [38,52,53,56,57,58] showed that the overlapping of Si–O/P–O bands could be an indication of Si incorporation into HAp structure by PO_4_^3−^ with SiO_4_^4−^substitution.

### 3.4. Biofilm Thickness and Cell Density Analysis Using Confocal Laser Scanning Microscopy (CLSM)

One of the goals of this research was to evaluate the antifungal activity of complex layers on *Candida* biofilms. Fungal strains resistant to antifungal drugs have become a major issue since they lead to increased fungal infections among people with lowered immunity such as HIV patients. Prostheses and implants can be considered sources with high potential for infection by oral candidiasis. Salvi *et al.* [67] in their studies on bacterial colonization patterns of *Staphylococcus aureus* and other bacteria such as *C. albicans* in implants and adjacent teeth showed that prostheses and implants can be considered sources with high potential for infection by oral candidiasis. According to Tobudic *et al.* [68], the flexibility in binding to the surfaces of the biomaterial and forming biofilms on its surface when they are in contact with the mucosa contribute to the virulence of *C. albicans*. Several studies including those developed by Penk and Pittrow [69] on the “role of fluconazole in the long-term suppressive therapy of fungal infections in patients with artificial implants”, declared that *C. albicans* can easily colonize and aggregate on prostheses and implants. More than that, Ramage *et al.* [70] in the study on “*Candida* biofilms on implanted biomaterials: a clinically significant problem” showed that the development of *Candida* biofilm on implantable materials is a major clinical problem.

More effective studies on the adhesion control of microbial biofilm on surfaces that may be used as coatings for implantable materials could lead to possible solutions both for preventing infections and for healing them. Our studies aim to highlight the bioactivity of composite layers based on zinc or silver doped hydroxyapatite that could be successfully used for coverage of implantable materials to decrease the risk of postoperative infections. The results of this study could have a major influence on the process of osseointegration of different implants.

**Figure 9:**
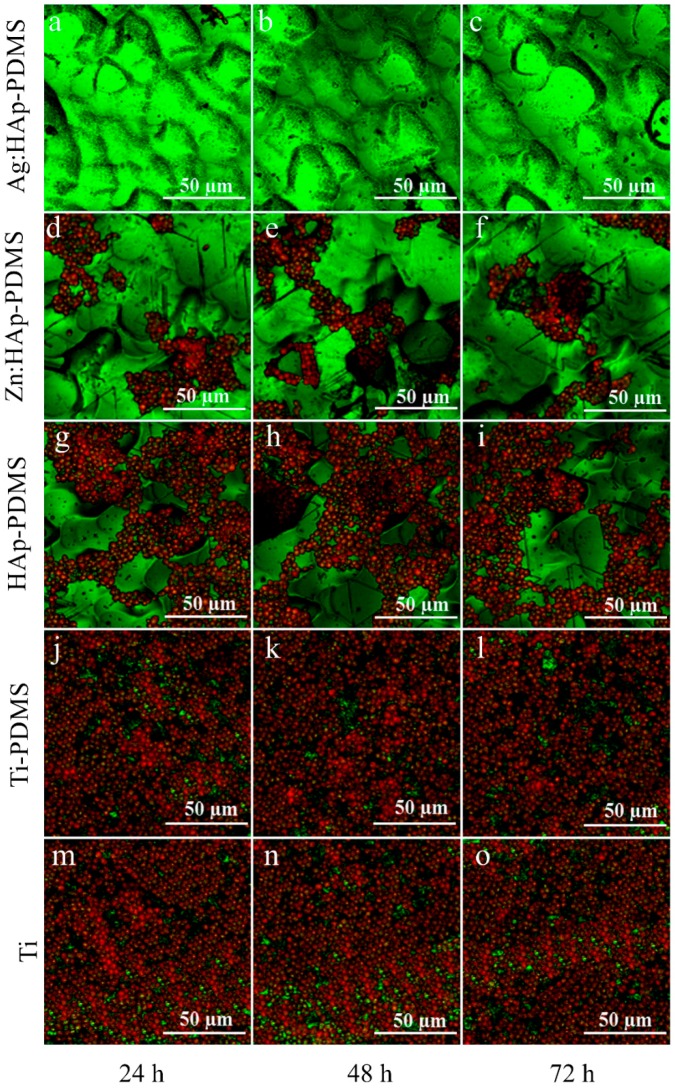
CLSM of biofilm image of *C. albicans* biofilm stained with propidium iodide developed on different substrates at various time intervals (**a**) biofilm structure developed on the Ag:HAp-PDMS after 24 h; (**b**) biofilm structure developed on the Ag:HAp-PDMS after 48 h; (**c**) biofilm structure developed on the Ag:HAp-PDMS after 72 h; (**d**) biofilm structure developed on the Zn:HAp-PDMS after 24 h; (**e**) biofilm structure developed on the Zn:HAp-PDMS after 48 h; (**f**) biofilm structure developed on the Zn:HAp-PDMS after 72 h; (**g**) biofilm structure developed on the HAp-PDMS after 24 h; (**h**) biofilm structure developed on the HAp-PDMS after 48 h; (**i**) biofilm structure developed on the HAp-PDMS after 72 h; (**j**) biofilm structure developed on the Ti-PDMS after 24 h; (**k**) biofilm structure developed on the Ti-PDMS after 48 h; (**l**) biofilm structure developed on the Ti-PDMS after 72 h; (**m**) biofilm structure developed on the Ti after 24 h; (**n**) biofilm structure developed on the Ti after 48 h; (**o**) biofilm structure developed on the Ti after 72 h. Red: live cells in the *C. albicans* biofilm; green: dead cells in the *C. albicans* biofilm.

The architecture of *C. albicans* biofilm was examined by CLSM and SEM after 24, 48 and 72 h from their development on various surfaces (Ti, PDMS, HAp-PDMS, Zn:HAp-PDMS and Ag:HAp-PDMS). Figure 9 shows the CLSM biofilm image of *C. albicans* grown on different surfaces. For CLSM imaging, the propidium iodide was used as marker for the labelling of bacterial cells in the biofilm (red). The CLSM of biofilm image revealed that the *C. albicans* biofilm stained in red by the propidium iodide developed very well on the Ti substrate, PDMS and HAp-PDMS composite layers at 24 h (Figure 9g,j,m), 48 h (Figure 9h,k,n) and 72 h (Figure 9i,l,o). The individual fungal cells were noticed on the Ti substrate (considered as a control) and the layers of PDMS (titanium substrate previously coated with PDMS layer) and HAp-PDMS (HAp nanoparticles deposited on a titanium substrate previously coated with a PDMS layer). The fungal cells observed in the CLSM images had almost round or slightly oval morphology, which is typical for *C. albicans* culture. The observations of CLSM images showed a well-structured cellular organization. The *C. albicans* cells on the surface of Zn:HAp-PDMS (zinc doped HAp on a Ti substrate previously coated with a PDMS layer) composite layer are in smaller number compared to controls at all investigated time intervals (Figure 9d–f). On the other hand, a slight decrease in the biofilm development after 48 and 72 h was evidenced. Contrariwise, it was noticed that the biofilm structure developed on the Ag:HAp-PDMS (silver doped HAp on a titanium substrate previously coated with a PDMS layer) composite layer showed an drastically decrease in biofilm development after 24 h (Figure 9a). Moreover, the biofilm images captured after 48 and 72 h revealed the absence of *C. albicans* cells (Figure 9b,c).

**Figure 10:**
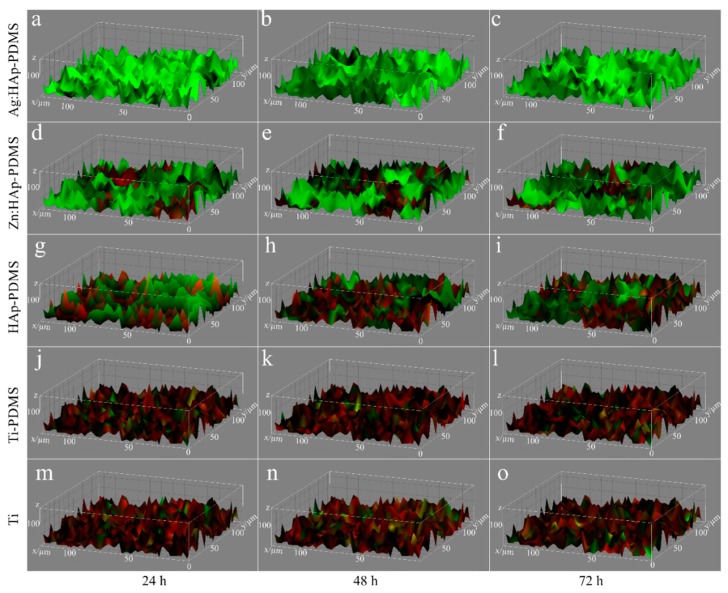
The 3D composite images of the *C. albicans* biofilm formation on various substrates after different incubation intervals (**a**) biofilm formed on the surface of Ag:HAp-PDMS composite layer after 24 h; (**b**) biofilm formed on the surface of Ag:HAp-PDMS composite layer after 48 h; (**c**) biofilm formed on the surface of Ag:HAp-PDMS composite layer after 72 h; (**d**) biofilm formed on the surface of Zn:HAp-PDMS composite layer after 24 h; (**e**) biofilm formed on the surface of Zn:HAp-PDMS composite layer after 48 h; (**f**) biofilm formed on the surface of Zn:HAp-PDMS composite layer after 72 h; (**g**) biofilm formed on the surface of HAp-PDMS composite layer after 24 h; (**h**) biofilm formed on the surface of HAp-PDMS composite layer after 48 h; (**i**) biofilm formed on the surface of HAp-PDMS composite layer after 72 h; (**j**) biofilm formed on the surface of Ti-PDMS composite layer after 24 h; (**k**) biofilm formed on the surface of Ti-PDMS composite layer after 48 h; (**l**) biofilm formed on the surface of Ti-PDMS composite layer after 72 h; (**m**) biofilm formed on the surface of Ti composite layer after 24 h; (**n**) biofilm formed on the surface of Ti composite layer after 48 h; (**o**) biofilm formed on the surface of Ti composite layer after 72 h. Red: live cells; green: dead cells.

To characterize the structure and spatial distribution of the live cells in the biofilm on the different surfaces (Ti, PDMS, HAp-PDMS, Zn:HAp-PDMS and Ag:HAp-PDMS) at 24, 48 and 72 h 3D composite images were analyzed using Image J software (Figure 10). The 3D image was made up of several biofilm layers along the y-axis. Given the thickness of the biofilm, by Image J software we could obtain automatically the dimensions of the *x*- and *y*-axis. On the other hand, the *z*-axis measures the fluorescence intensity of live (red color) cells. In the pictures shown in Figure 10 is illustrated the spatial distribution of the live (red color) cells in the *C. albicans* biofilm along horizontal (coverage) and vertical (thickness) distributions on the different substrate (Ti, PDMS, HAp-PDMS, Zn:HAp-PDMS and Ag:HAp-PDMS) at different time intervals (24, 48 and 72 h) as indicated by the fluorescence intensity.

The results revealed that the *C. albicans* biofilm structure and composition were similar on Ti substrate (considered as a control), PDMS and HAp-PDMS composite layers regardless of the studied time interval. Nevertheless, live cells (red color) are no longer dominant in the biofilm formed on the surface of Zn:HAp-PDMS composite layer. One can also notice that the evolution of *C. albicans* biofilm on Ti substrate coated with Zn:HAp-PDMS composite layer was slightly diminished over time (Figure 10e,f). The fungicidal effect [71] of the surface Ag:HAp-PDMS composite layer on biofilm formation and development was shown against *C. albicans* (Figure 10a–c).

### 3.5. Biofilm Morphology Analysis Using Scanning Electron Microcopy (SEM)

**Figure 11:**
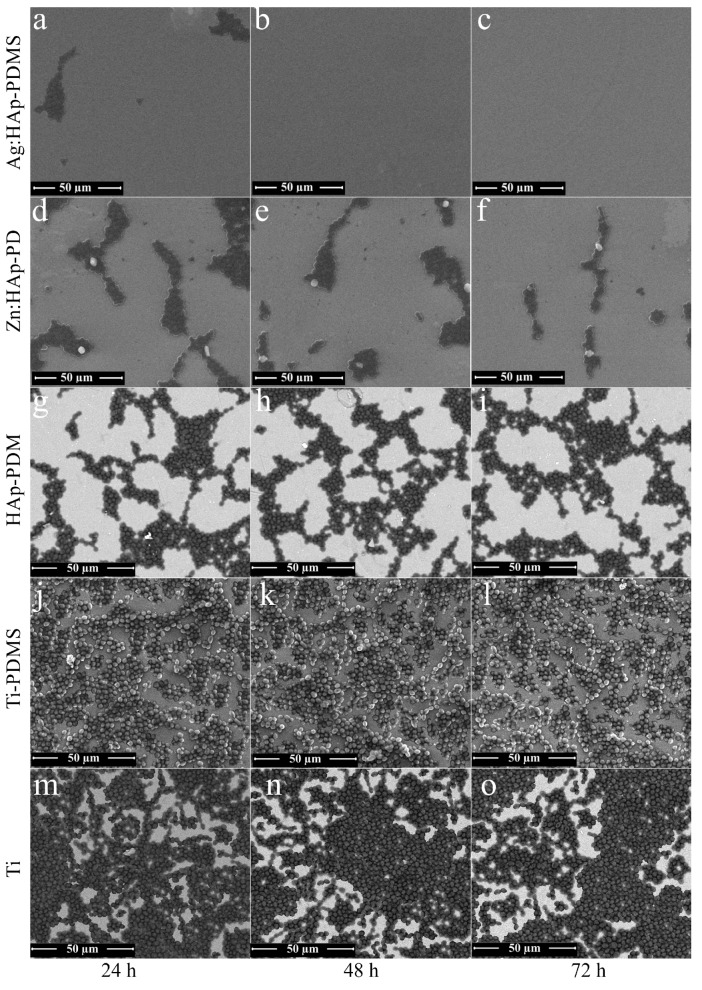
SEM images with a lower magnification (5000×) of *C. albicans* biofilm stained with propidium iodide developed on different substrates at various time intervals (**a**) cell organization in *C. albicans* biofilm on the surface of Ag:HAp-PDMS composite layers after 24 h; (**b**) cell organization in *C. albicans* biofilm on the surface of Ag:HAp-PDMS composite layers after 48 h; (**c**) cell organization in *C. albicans* biofilm on the surface of Ag:HAp-PDMS composite layers after 72 h; (**d**) cell organization in *C. albicans* biofilm on the surface of Zn:HAp-PDMS composite layers after 24 h; (**e**) cell organization in *C. albicans* biofilm on the surface of Zn:HAp-PDMS composite layers after 48 h; (**f**) cell organization in *C. albicans* biofilm on the surface of Zn:HAp-PDMS composite layers after 72 h; (**g**) cell organization in *C. albicans* biofilm on the surface of HAp-PDMS composite layers after 24 h; (**h**) cell organization in *C. albicans* biofilm on the surface of HAp-PDMS composite layers after 48 h; (**i**) cell organization in *C. albicans* biofilm on the surface of HAp-PDMS composite layers after 72 h; (**j**) cell organization in *C. albicans* biofilm on the surface of Ti-PDMS composite layers after 24 h; (**k**) cell organization in *C. albicans* biofilm on the surface of Ti-PDMS composite layers after 48 h; (**l**) cell organization in *C. albicans* biofilm on the surface of Ti-PDMS composite layers after 72 h; (**m**) cell organization in *C. albicans* biofilm on the surface of Ti-PDMS composite layers after 24 h; (**n**) cell organization in *C. albicans* biofilm on the surface of Ti-PDMS composite layers after 48 h; (**o**) cell organization in *C. albicans* biofilm on the surface of Ti-PDMS composite layers after 72 h.

To go further in the presentation of cell organization in *C. albicans* biofilm, representative SEM images of the surface biofilm were collected at 24, 48 and 72 h (Figure 11). 

SEM examination allowed observing the biofilm morphology at the cellular level for each sampling time. SEM observations have revealed the individual fungal cells on the surface of control (Ti substrate) at 24, 48 and 72 h (Figure 11m–o). The same comportment of *C. albicans* biofilm developed on the surface of PDMS composite layers was likewise observed at different times (Figure 11j,k). Moreover, the behavior of *C. albicans* biofilm developed on the surface of HAp-PDMS composite layers was also similar at different times (Figure 11g–i).The morphology of *C. albicans* fungal cells observed from SEM images is typical of *C. albicans* culture.

SEM image showed that the *C. albicans* cells were almost round or slightly oval. The *C. albicans* biofilm revealed different growth rates on the Zn:HAp-PDMS composite layers relative to the control. A decrease in *C. albicans* biofilm development on the surface of Zn:HAp-PDMS composite layer was also observed when the incubation time increased (Figure 11d–f). 72 h after the incubation, the *C. albicans* biofilm growth rate on the Zn:HAp-PDMS composite layer was drastically diminished (Figure 11f). The antifungal properties of Ag:HAp-PDMS composite layers against the *C. albicans* biofilm was evidenced in Figure 11a–c. For a 24 h incubation time, the fungi static effect of Ag:HAp-PDMS composite layers against *C. albicans* biofilm was observed (Figure 11a). Moreover, after an incubation time of 48 and 72 h, the fungicidal effects of Ag:HAp-PDMS composite layers against *C. albicans* biofilm was highlighted (Figure 11b,c). The results obtained from SEM investigations come to confirm the CLSM observations.

According to previous studies on “Ceramic/metal biocidal nanocomposites for bone-related applications”, reported by Miranda *et al.* [72], nanoparticles can attach to the cell membrane, causing structural changes that can lead to cell death. More than that, recent studies demonstrated that both free radical formation [73] and release of ions by nanoparticles can contribute to antimicrobial activity [74]. Morones *et al.* [75] in their study showed that the antimicrobial effects of composite materials based on silver are caused by the gradual release of silver ions. Furthermore, in the studies recently reported [46,76,77,78] on silver doped hydroxyapatite nanoparticles, we evaluated the antibacterial and antifungal activities. Following the studies previously conducted [46,77,78], we noticed an antimicrobial effect against various Gram-negative and Gram-positive bacteria and also against fungal species such as *Candida krusei*. On the other hand, our present results are in good agreement with the previous results conducted by Martinez-Gutierrez *et al.* [78] which have shown that the silver nanoparticles inhibit the biofilm formation and as a result of the gradual release, the silver ions can cause an increase of the antimicrobial effects duration. Besides, Zamperini *et al.* [79] evaluated the effects of the silver doped hydroxyapatite solution against the biofilm formation of *C. albicans*. According to results recently presented by De Silva *et al.* [80] the antimicrobial effect of various zinc-based composites could be caused by the presence of Zn^2+^ ions, and by the generation of reactive oxygen species as a result of photocatalytic activities. Our studies on antifungal properties of Zn:HAp-PDMS composite layers against the *C. albicans* biofilm, exhibited in this work confirmed the previous results obtained by De Silva *et al.* [80].

In the studies on “‘Nanoantibiotics’: A new paradigm for treating infectious diseases using nanomaterials in the antibiotics resistant era” [81], Huh A.J. and Kwon Y.J. showed that, despite the fact that nanoparticle antimicrobial mechanisms are not well understood, it is assumed that when they come into contact with cells, they provoke the production of reactive oxygen species (ROS), cell membrane disruption, mitochondrial damage and DNA damage. Furthermore, the structural studies on the microbial interaction with nanostructured material could provide new information for better understanding the influence of nanoparticles. On the other hand, the efficacy of nanoparticles against bacteria and fungi was demonstrated in several studies [72,73,74,75,76,77,78,79,80,81]. It was shown that the nanoparticles accumulate in the bacterial membrane, and some of them penetrate into the cells [82]. More than that, it was appreciated that the nanoparticles induced transformations especially in the cell membrane morphology, causing a significant increase in their permeability, thus affecting the proper transport through the plasma membrane and, ultimately, leading in cell death. Furthermore, Le A.T. *et al.* [83] in their evaluations on "powerful colloidal silver nanoparticles for the prevention of gastrointestinal bacterial infections", showed that the nanoparticles with small diameters penetrated the cell walls.

To summarize, this study presented the use of different composite layers as a strategy for retardation or prevention of *C. albiacans* biofilm formation. Our studies come to confirm the fungicidal effect of silver. In addition, our results suggest that Ag:HAp-PDMS surface may be used for medical treatment of biofilm associated *Candida* infections. We also showed that the Zn:HAp-PDMS surface has antifungal properties against *C. albicans*. The use of these materials could have a major impact in medical applications because they could help prevent infections. Applying these layers on different medical instruments may lead to an increase of their life time, thus lowering the costs related to hospital care.

Additionally, further research will involve clinical and microbiological studies on ZnHAp-PDMS and Ag:HAp-PDMS complex layers for a careful assessment regarding their antifungal properties considering that no concrete and accurate data on these properties are yet available. Moreover, *in vivo* studies on animal subjects need to be conducted.

## 4. Conclusions

The present study presents the results of the surface and structural characterization of Ag:HAp-PDMS, Zn:HAp-PDMS and HAp-PDMS complex layers deposited on Ti substrates. The morphology of these coatings was investigated by Scanning Electron Microscopy and a 3D plot of the surfaces was also presented. SEM micrographs were also used to highlight the influence of the PDMS polymer interlayer. The obtained results suggested that the use of a polymer interlayer for the HAp coating could overcome its delamination even when cracks appear. When a rough Ti surface was used as substrate, it was observed that the polymer covers uniformly the uneven substrate surface, promoting the Zn:HAp coating adherence to the rough substrate. It was observed that even if the coating is non-uniform, delaminated and cracked, its embedding into the polymer could prevent it from further deterioration. Further analysis of the constituent elements distributed throughout the coatings and of the depth profiles of the thin films were performed by GDOES measurements. The Si, Ca and P depth profile curves begin and ended simultaneously, thus indicating that the HAp, Ag:HAp and Zn:HAp respectively were successfully embedded into the polymer. Furthermore, the GD depth profiles indicate the formation of a composite material. Additional structural characterization of the samples was provided by FTIR measurements. The IR spectra of the HAp-PDMS, Ag:HAp-PDMS and Zn:HAp-PDMS layers and the associated second derivatives were studied and interpreted. All the functional groups characteristic to both the apatitic and PDMS structures were found in the IR spectra. The results also revealed that on the substrates partially covered with a PDMS layer, some SiO_4_^4−^ groups were present. As a result of the thermal treatment performed on the samples after the deposition of HAp, Ag:HAp and Zn:HAp on the titanium substrates previously coated with the PDMS layer, some substitutions of the PO_4_^3−^ from the apatitic structure by SiO_4_^4−^ were possible. The second derivative analysis also revealed the peaks characteristic to the SiO_4_^4−^ functional group along with the other peaks associated to the apatitic and PDMS structures.

On the other hand, *in vitro* evaluation of the antifungal properties of Ag:HAp-PDMS and Zn:HAp-PDMS composite layers was established. It was distinctly demonstrated the fungicidal effects of Ag:HAp-PDMS composite layers against *C. albicans* biofilm and the potential antifungal effect of Zn:HAp-PDMS composite layers against *C. albicans* biofilm.

The results acquired in this research complete previous research on the potential use of new complex materials produced by nanotechnology in biomedicine. Our hope is that our research will constitute the premises for the future of the biomedical field.

## Figures and Tables

**Table 1:** IR vibrational bands observed in the FT-IR spectra of HAp-PDMS, Ag:HAp-PDMS and Zn:HAp-PDMS composite layers.

IR Bands Assigment	HAp-PDMS Wavenumber (cm^−1^)	Ag:HAp-PDMS Wavenumber (cm^−1^)	Zn:HAp-PDMS Wavenumber (cm^−1^)
SiO_4_^4−^	498	489	511
ν_4_ PO_4_^3−^	600	570	567
C–H in Si–CH_3_	701	737	–
Si(CH_3_)_2_	–	–	619
Si–O in SiO_2_	794	775	774
Si–C/Si–O	862	857	852
Si–O–Si in siloxane	1,014	984	989
ν_3_ PO_4_^3−^	–	–	1,053
Si–O–Si siloxane	1,086	1,070	–
C–H bond in Si=CH_3_ group	1,260	1,255	1,252
	1,413	1,413	1,397
H_2_O, OH, Si–OH	1,650	1,655	1,600

## Abbreviations

The following abbreviations are used in this manuscript:

PDMS	Poly(dimethylsiloxane)
Ti	Titanium
HAp	Hydroxyapatite
Ag:HAp	Silver doped hydroxyapatite
Zn:HAp	Zinc doped hydroxyapatite
HAp-PDMS	Hydroxyapatite on a Titanium substrate previously coated with polydimethylsiloxane
Ag:HAp-PDMS	Silver doped hydroxyapatite on a Titanium substrate previously coated with polydimethylsiloxane
Zn:HAp-PDMS	Zinc doped hydroxyapatite on a Titanium substrate previously coated with polydimethylsiloxane
SEM	Scanning Electron Microscopy
GDOES	Glow Discharge Optical Emission Spectroscopy
GD	Glow Discharge
FTIR	Fourier Transform Infrared Spectroscopy
YPG	Yeast peptone glucose medium
CLSM	Confocal laser scanning microcopy
R	Surface roughness
IR	Infrared
